# Cardiomegaly and Low Voltage Suggest Cardiac Involvement in a Patient With Hypereosinophilic Syndrome: A Case Report

**DOI:** 10.7759/cureus.39354

**Published:** 2023-05-22

**Authors:** Katsuro Kashima, Masaomi Ooi, Yusuke Yoshishige, Kazuyo Kawabata

**Affiliations:** 1 Cardiology, National Hospital Organization Ibusuki Medical Center, Ibusuki, JPN

**Keywords:** hypereosinophilic syndrome, eosinophilic myocarditis, fulminant necrotizing eosinophilic myocarditis, peripheral blood eosinophil count, endomyocarditis, decompensated heart failure

## Abstract

Hypereosinophilic syndrome is a heterogeneous group of disorders, the majority of which are idiopathic. Cardiac manifestations, particularly eosinophilic myocarditis and endomyocardial fibrosis, are a typical course of morbidity and mortality in hypereosinophilic syndrome. We present a case of a patient with asthma and idiopathic eosinophilia who presented with dyspnea and edema. Cardiac ultrasonography showed pericardial effusion and reduced left ventricular motion, which persisted despite heart failure therapy, although pulmonary congestion improved. The peripheral blood eosinophil count was markedly elevated four days after admission, even though eosinophilia was not present at admission. Parasitic disease, autoimmune disease, and drug-induced cardiomyopathy were excluded as possibilities. A high dose of steroid therapy was started due to eosinophilic myocarditis. Cardiac function improved soon after therapy, along with a reduction in eosinophils. Upon retrospective examination, cardiomegaly and low voltage were observed, along with an elevation in the eosinophil count 15 months before admission. Monitoring chest radiography and electrocardiograms according to fluctuations in eosinophils may enable early detection and treatment of cardiac involvement in patients with hypereosinophilic syndrome, as demonstrated by this case.

## Introduction

Hypereosinophilic syndrome (HES) is a rare and heterogeneous group of disorders characterized by persistent eosinophilia (>1.5 × 109/L eosinophils), evidence of eosinophil-associated end-organ damage, and the absence of a secondary cause [1,2]. Cardiac dysfunction is a common complication of HES and is a major cause of morbidity and mortality [3,4]. However, diagnosing HES can be challenging, especially in the absence of peripheral eosinophilia [5-7].

Here, we report a case of heart failure with hypereosinophilia in which eosinophilia was not present upon admission but developed several days later. The patient exhibited cardiomegaly on chest radiography and a low voltage on the electrocardiogram, which fluctuated in response to changes in the eosinophil count before hospitalization. These findings suggest the presence of an underlying cardiac abnormality prior to the onset of congestive heart failure. It is important to recognize and treat cardiac involvement early in the course of HES to improve outcomes.

## Case presentation

**Figure 1:**
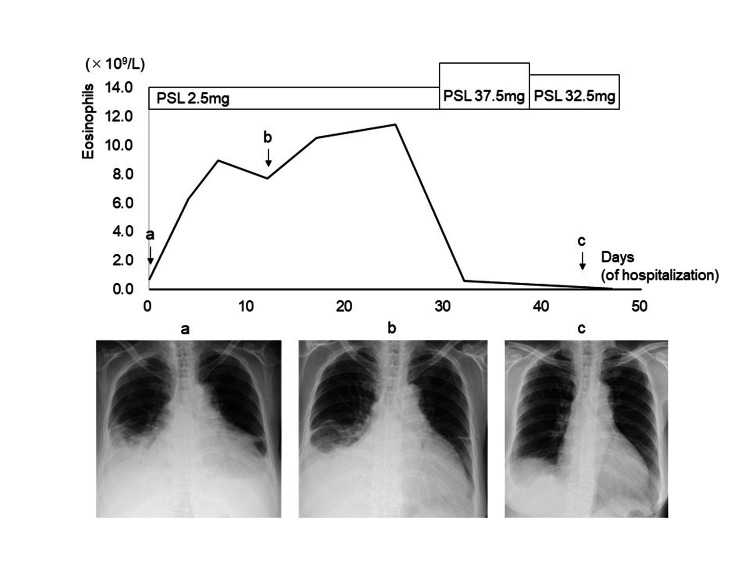
Clinical course after hospitalization. Panel (A) shows the chest radiography at admission displaying bilateral pleural effusion and cardiomegaly. Four days after admission, the eosinophil count in peripheral blood was found to be markedly elevated, and despite therapy for heart failure, sustained cardiomegaly and pleural effusion were observed on chest radiography (panel B). However, the eosinophil count immediately decreased after high-dose steroid therapy, leading to a dramatic improvement in cardiomegaly and pleural effusion (panel C). Prednisolone (PSL) was used for the therapy.

**Figure 2:**
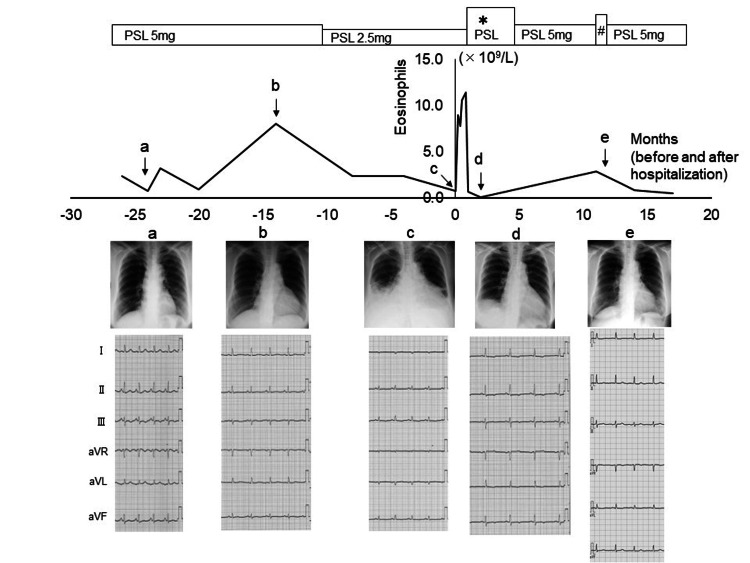
Clinical course before and after hospitalization. Panel (A) shows the chest radiography and an electrocardiogram during treatment with low-dose steroid therapy 24 months before admission, which were almost normal. However, cardiomegaly on chest radiography and a low voltage on an electrocardiogram were observed 14 months before admission, along with an elevation in the eosinophil count (panel B). At admission, cardiomegaly and the low voltage worsened, although eosinophilia was absent (panel C). After high-dose steroid therapy, eosinophilia immediately decreased, and cardiomegaly, pleural effusion, and low voltage dramatically improved (panel D). Cardiomegaly further improved during outpatient care with prednisolone (PSL) at a maintenance dose of 5 mg/day (panel E). Note that the patient received PSL at a dose of 37.5 mg/day for 14 days, which was gradually tapered to a maintenance dose of 5 mg/day over the course of five months (shown as *). Additionally, 10 months later, the patient suffered from dyspnea along with an elevation in the eosinophil count, which was again treated with PSL (shown as #).

A 68-year-old woman was admitted to our hospital due to dyspnea and edema. She had a history of asthma and idiopathic eosinophilia over the past decade. However, despite previous treatment, she developed shortness of breath and lower leg edema. A chest X-ray revealed cardiomegaly and pleural effusion (Figure 1), and an electrocardiogram showed low voltage, abnormal Q waves, and sinus tachycardia (Figure 2C).

Echocardiography revealed reduced left ventricular wall motion. She had been on low-dose prednisolone for asthma and hypereosinophilia before admission. The cardiomegaly on the chest X-ray and voltage on the electrocardiogram had fluctuated with changes in eosinophil count (Figure 2).

Laboratory findings on admission showed markedly elevated brain natriuretic peptide concentration of 2710.3 pg/mL (normal range: <18.4 pg/mL), white blood cell count of 8.4 × 109/L (neutrophils: 6.3 × 109/L, eosinophils: 0.7 × 109/L), elevated C-reactive protein concentration of 6.2 mg/dL (normal range: <0.3 mg/dL), and increased troponin I concentration of 0.53 ng/mL (normal range: <0.03 ng/mL), suggesting inflammation and myocardial damage.

The patient was diagnosed with congestive heart failure and treated with diuretics, catecholamine, and carperitide, which provided relief from dyspnea but did not resolve cardiomegaly and pleural effusion. Therefore, a beta-blocker (bisoprolol 1.25 mg) was added. Eosinophil counts were elevated at 6.3 × 109/L (45.8%) four days after admission and further increased to 10.5 × 109/L (58.9%) 17 days after admission (Figure 1). Possible causes of eosinophilia were investigated, including parasitic and fungal diseases, medication or food allergies, autoimmune diseases, and tumors. The patient had no history of eating raw fish or meat, and serum parasite antigen tests were negative. Beta-D glucan, interferon-gamma release assays, anti-nuclear antibody, and P-antineutrophil cytoplasmic antibody were also negative, and pleural effusion showed no abnormalities.

Echocardiography revealed severe systolic dysfunction with an ejection fraction of 24%, and no thickening of the ventricular wall, apical thrombus, or posterior mitral leaflet involvement was observed. On day 20, cardiac catheterization showed a pulmonary artery systolic pressure of 45 mmHg, pulmonary wedge pressure of 17 mmHg, right atrial pressure of 7 mmHg, and cardiac index of 2.41 L/min/m^2^. Coronary arteries showed no atherosclerotic changes. Left ventriculography showed severely reduced wall motion, with a diastolic volume of 225 mL, systolic volume of 170 mL, and ejection fraction of 24%.

The patient's hypereosinophilia persisted even after stopping medications taken before admission, and she did not recover from congestion. Therefore, she was clinically diagnosed with heart failure accompanied by eosinophilic myocarditis without undergoing a myocardial biopsy. On day 31, she was treated with oral prednisolone 37.5 mg/day (0.75 mg/kg) for 14 days, after which the dose was tapered to 32.5 mg/day. The patient’s eosinophil count decreased rapidly to 0.6 × 109/L (4.8%) the day after receiving high-dose steroid therapy, and their cardiac function improved concurrently with a decrease in eosinophils (Figure 1).

Following the successful tapering of prednisolone to a maintenance dose of 5 mg/day, the patient was discharged and monitored as an outpatient by tracking their eosinophil count. Ten months later, the patient experienced dyspnea and an increase in eosinophil count to 2.8 × 109/L (19%) and was treated with prednisolone at a dose of 30 mg/day for seven days. The dose was then reduced to 10 mg/day and gradually tapered down to a maintenance dose of 5 mg/day (Figure 2). Presently, the patient’s prednisolone dose is 2 mg/day, and she remains asymptomatic without cardiomegaly for seven years.

## Discussion

Hypereosinophilia is characterized by an elevated eosinophil count (>1.5 × 109/L) observed on two separate tests more than one month apart. HES is a rare group of disorders that result in peripheral hypereosinophilia and organ damage directly attributed to tissue hypereosinophilia. Cardiac manifestations, particularly eosinophilic myocarditis and endomyocardial fibrosis, are common causes of morbidity and mortality in HES [1,2,8]. Loeffler first reported the association between eosinophilia and heart disease in his observation of fibroplastic parietal endocarditis and peripheral eosinophilia [9]. The cardiac pathology of HES can be classified into three stages: an acute necrotic stage, a thrombotic-necrotic stage, and a late fibrotic stage [2]. Although the precise pathogenic mechanism of acute necrotizing eosinophilic myocarditis has not been defined, extensive infiltration of eosinophils into the myocardium and activated eosinophils may cause tissue damage through the release of toxic granules, cytokines, or recruitment of inflammatory cells [8].

Cardiac magnetic resonance imaging can detect endocardial fibrosis with wall thickness indicating inflammatory edema, although this technique was not available in our case. While an endomyocardial biopsy is the gold standard for definitive diagnosis of eosinophilic endomyocardial disease, biopsies can cause ventricular perforation, arrhythmia, conduction abnormalities, and considerable sampling error related to patchy or focally distributed myocarditis. We could not perform a cardiac biopsy because our patient did not provide approval.

During the initial stage of cardiac involvement, echocardiography is typically normal, although wall thickening may be identified if there is considerable edema within the myocardium because of the inflammatory process. In the thrombotic stage, thrombi can be identified within both apices of the ventricles. In the late stage, echocardiography can reveal endomyocardial and valvular tissue fibrosis, which may lead to mitral regurgitation and restrictive cardiomyopathy [2,10-12]. However, these findings were not present in our case.

In most cases of eosinophilic myocarditis, peripheral eosinophils increase. However, eosinophilia was not present at the time of the onset of heart failure in this case. In some patients with eosinophilic myocarditis, eosinophilia was not present at the time of admission [13], and in other cases, the eosinophil count was markedly elevated several days after admission. Consistent monitoring of the eosinophil count in peripheral blood is important in cases of heart failure of an unknown etiology. Some cases of eosinophilic myocarditis without peripheral eosinophilia were diagnosed by endomyocardial biopsy [5,7], and prednisolone therapy was effective in most cases of eosinophilic myocarditis [14]. Although a cardiac biopsy was not performed in our case, we excluded parasitic disease, autoimmune disease, and drug-induced cardiomyopathy.

Echocardiography showed severe systolic dysfunction, and serological findings suggested inflammation and myocardial damage. Therefore, we diagnosed our patient with eosinophilic myocarditis because her clinical features conformed to the diagnostic criteria of the guideline of the Japanese Circulation Society [15]. Her eosinophil count immediately decreased after high-dose steroid therapy, and then her cardiomegaly and pleural effusion dramatically improved.

In the present case, the patient had received low-dose prednisolone for asthma and idiopathic hypereosinophilia before being admitted to the hospital. Cardiomegaly was evident on chest radiography and electrocardiogram voltage varied in response to changes in eosinophil count, indicating a potential underlying cardiac abnormality before the onset of congestive heart failure. However, no detailed studies have focused on the relationship between fluctuation in peripheral eosinophils and cardiomegaly with low voltage before eosinophilic myocarditis sets in. Upon retrospective examination of this case, cardiomegaly and low voltage were observed with an elevation in eosinophil count 15 months prior to admission. The patient was followed up successfully in outpatient care for over seven years after hospitalization to confirm the presence or absence of cardiomegaly, low voltage, and eosinophil count.

These findings underscore the importance of early recognition of cardiac involvement in patients with HES, which can be evaluated using simple and cost-effective diagnostic equipment as part of the initial test.

## Conclusions

HES can lead to decompensated heart failure, and it is crucial to regularly measure the eosinophil count in peripheral blood. If a significantly elevated eosinophil count is detected, cardiac involvement must be assessed, and routine chest radiography and electrocardiogram have the potential to avoid overlooking cardiac abnormality in patients with idiopathic eosinophilia. Early detection and prompt initiation of treatment for heart failure may prevent its progression to a severe stage.

## References

[1] Ogbogu PU, Bochner BS, Butterfield JH (2009). Hypereosinophilic syndrome: a multicenter, retrospective analysis of clinical characteristics and response to therapy. J Allergy Clin Immunol.

[2] Mankad R, Bonnichsen C, Mankad S (2016). Hypereosinophilic syndrome: cardiac diagnosis and management. Heart.

[3] Amini R, Nielsen C (2010). Eosinophilic myocarditis mimicking acute coronary syndrome secondary to idiopathic hypereosinophilic syndrome: a case report. J Med Case Rep.

[4] Lim J, Sternberg A, Manghat N, Ramcharitar S (2013). Hypereosinophilic syndrome masquerading as a myocardial infarction causing decompensated heart failure. BMC Cardiovasc Disord.

[5] Watanabe N, Nakagawa S, Fukunaga T, Fukuoka S, Hatakeyama K, Hayashi T (2001). Acute necrotizing eosinophilic myocarditis successfully treated by high dose methylprednisolone. Jpn Circ J.

[6] Getz MA, Subramanian R, Logemann T, Ballantyne F (1991). Acute necrotizing eosinophilic myocarditis as a manifestation of severe hypersensitivity myocarditis. Antemortem diagnosis and successful treatment. Ann Intern Med.

[7] Sugiyama E, Takenaka T, Kato M (2015). Eosinophilic myocarditis without hypereosinophilia accompanied by giant cell infiltration. J Cardiol Cases.

[8] deMello DE, Liapis H, Jureidini S, Nouri S, Kephart GM, Gleich GJ (1990). Cardiac localization of eosinophil-granule major basic protein in acute necrotizing myocarditis. N Engl J Med.

[9] Loeffler W (1936). Endocarditis parietalis fibroplastica mit Bluteosinophilie, ein eigenartiges Krankheitsbild. Schweiz Med Wochenschr.

[10] Ommen SR, Seward JB, Tajik AJ (2000). Clinical and echocardiographic features of hypereosinophilic syndromes. Am J Cardiol.

[11] Arustamyan M, Hoosain J, Mattson J, Hasni SF, Cho SH, Gorodin Kiliddar P (2019). Loeffler endocarditis: a manifestation of hypereosinophilic syndrome. CASE (Phila).

[12] Niemeijer ND, van Daele PL, Caliskan K, Oei FB, Loosveld OJ, van der Meer NJ (2012). Löffler endocarditis: a rare cause of acute cardiac failure. J Cardiothorac Surg.

[13] Morimoto S, Kubo N, Hiramitsu S (2003). Changes in the peripheral eosinophil count in patients with acute eosinophilic myocarditis. Heart Vessels.

[14] Kawano S, Kato J, Kawano N (2011). Clinical features and outcomes of eosinophilic myocarditis patients treated with prednisolone at a single institution over a 27-year period. Intern Med.

[15] JCS Joint Working Group (2011). Guidelines for diagnosis and treatment of myocarditis (JCS 2009): digest version. Circ J.

